# Isolation of Porcine Adenovirus Serotype 5 and Construction of Recombinant Virus as a Vector Platform for Vaccine Development

**DOI:** 10.3390/v17091270

**Published:** 2025-09-19

**Authors:** Qianhua He, Jun Wu, Zhilong Bian, Yuan Sun, Jingyun Ma

**Affiliations:** 1 State Key Laboratory of Swine and Poultry Breeding Industry, South China Agricultural University, Guangzhou 510642, China; heqianhua11@stu.scau.edu.cn (Q.H.); wjun0075@163.com (J.W.); sunyuan@scau.edu.cn (Y.S.); 2 Artemis Shield Animal Health Co., Ltd., Guangzhou 510000, China; zhilongforever@sina.com

**Keywords:** porcine adenovirus serotype 5, Reverse genetics Viral vector, E3 region vaccine platforms

## Abstract

Porcine adenovirus serotype 5 (PAdV-5) is an emerging viral vector platform for veterinary vaccines; however, its genomic plasticity and essential replication elements remain incompletely characterized. This study reports the isolation and reverse genetic manipulation of a novel PAdV-5 strain (GD84) from diarrheic piglets in China. PCR screening of 167 clinical samples revealed a PAdV-5 detection rate of 38.3% (64/167), with successful isolation on ST cells after three blind passages. The complete GD84 genome is 32,620 bp in length and exhibited 99.0% nucleotide identity to the contemporary strain Ino5, but only 97.0% to the prototype HNF-70. It features an atypical GC content of 51.0% and divergent structural genes—most notably the hexon gene (89% identity to HNF-70)—suggesting altered immunogenicity. Using Red/ET recombineering, we established a rapid (less than 3 weeks) reverse genetics platform and generated four E3-modified recombinants: ΔE3-All-eGFP, ΔE3-12.5K-eGFP, ΔE3-12.5K+ORF4-eGFP, and E3-Insert-eGFP. Crucially, the ΔE3-All-eGFP construct (complete E3 deletion) failed to be rescued, while constructs preserving the 12.5K open reading frame (ORF) yielded replication-competent viruses with sustained eGFP expression over three serial passages and titers over 10^7.0^ TCID_50_/mL. Fluorescence intensity was inversely correlated with genome size, as the full-length E3-Insert-eGFP virus showed reduced expression compared with the ΔE3 variants. Our work identifies the 12.5K ORF as essential for PAdV-5 replication and provides an optimized vaccine engineering platform that balances genomic payload capacity with replicative fitness.

## 1. Introduction

Adenoviruses, first isolated in human adenoid tissue in 1953, have since been established as non-enveloped viruses with a linear double-stranded DNA genome (~26–48 kb) encapsidated in an icosahedral capsid [1]. Their structural robustness, coupled with efficient transduction of dividing and non-dividing cells, has rendered them pivotal vectors for gene therapy and vaccinology [2]. The genome organization, featuring early (E1–E4) and late (L1–L5) transcriptional regions—enables precise genetic manipulation, particularly through E1/E3 deletions to generate replication-defective vectors [2,3]. The fiber knob domain dictates species-specific tropism by binding cellular receptors like CAR (coxsackievirus and adenovirus receptor), while penton base proteins mediate internalization via integrins [4]. This molecular plasticity has positioned adenoviruses as pioneering vectors for gene therapy and vaccines.

Based on phylogenetic analysis, adenoviruses are divided into six genera, Atadenovirus, Aviadenovirus, Ichtadenovirus, Mastadenovirus, Siadenovirus, and Testadenovirus [5]. Porcine adenoviruses (PAdVs), belonging to the genus Mastadenovirus, were identified as five immunologically distinct serotypes (PAdV-1 to 5) [5]. According to the demarcation, PAdVs were reclassified into mastadenovirus A, B, and C. PAdV-1 was first isolated from a rectal swab in 1964. This was followed by the isolation of PAdV-2 and PAdV-3 from healthy pigs, and subsequently PAdV-4 from the brain of a 10-month-old pig [6,7]. PAdV-5 (officially classified as *Porcine mastadenovirus C*) was first identified in 1987 from swine nasal swabs in Japan, with representative strains including HNF-61 and HNF-70 [8]. Phylogenetic studies indicate that PAdV-5 is more closely related to bovine adenoviruses than to human adenoviruses, suggesting possible cross-species evolutionary events [9].

PAdVs have garnered increasing interest both as potential pathogens in swine and as promising vectors for veterinary vaccines, particularly PAdV-3 and PAdV-5 [10]. Although human adenoviruses are well-characterized, key biological aspects of PAdV-5—including its genomic plasticity and mechanisms of host interaction—remain inadequately explored [11].

The E3 region of adenoviruses varies in length from 1 kb to 5.6 kb and encodes multiple overlapping mRNAs [12] that produce immunomodulatory proteins aiding in host immune evasion [13]. The potential of porcine adenoviruses, especially PAdV-3 and PAdV-5 as vaccine vectors, has been well demonstrated. Notable examples include: (i) a recombinant PAdV-3 vector expressing the classical swine fever virus (CSFV) E2 protein, which conferred 100% protection in challenge trials despite modest titers [14], and (ii) and (ii) an E3-modified PAdV-5 strain expressing the transmissible gastroenteritis virus (TGEV) S protein, which sustained stable antigen expression over serial passages and induced mucosal IgA responses [15]. These studies confirm the genomic flexibility and immunogenic potential of porcine adenovirus vectors. The large E3 insertion capacity of PAdV-5 (>2.3 kb) offers a particular advantage for multivalent vaccine design [16]. Earlier work indicated that at least 60% of the E3 region is non-essential for viral replication. A recombinant PAdV-5 with a partial E3 deletion retained viability and exhibited a genome size equivalent to 109.6% of the wild-type virus.

While reverse genetics systems for adenoviruses are well established, their application to porcine adenoviruses (PAdVs), particularly serotype 5 (PAdV-5), has been hampered by technical challenges related to their large genome size and the inefficiency of traditional construction methods. This study aimed to address these limitations by developing a rapid and highly efficient reverse genetics platform based on Red/ET recombineering in *E. coli*. We applied this system to a contemporary Chinese PAdV-5 field isolate (GD84) to systematically define the essential elements for viral replication and to establish design principles for next-generation vaccine vectors that balance transgene capacity with replicative fitness.

## 2. Materials and Methods

### 2.1. Virus Isolation

Fecal swab samples collected from diarrheic piglets in Guangdong, China were suspended in phosphate-buffered saline (PBS, Sangon Biotech, Shanghai, China) containing antibiotics (1000 IU/mL penicillin, 1000 µg/mL streptomycin, 50 µg/mL gentamicin, all from Sangon Biotech) to prepare a 10–20% (*w*/*v*) suspension. The suspension was vigorously vortexed and clarified by centrifugation at 10,000× *g* for 30 min at 4 °C. The resulting supernatant was filtered through a sterile 0.22 µm pore-size membrane filter (Millipore, Burlington, MA, USA) to remove bacteria and fungi. Aliquots of the filtered supernatant were either stored at −80 °C or used immediately for cell inoculation. For virus isolation, the porcine kidney (PK-15) and the swine testis (ST) cell lines have been maintained in our laboratory’s historical stock collection for an extended period. The cells were routinely cultured and authenticated based on morphological characteristics and were confirmed to be free of mycoplasma contamination prior to use. Cells were routinely cultured in Dulbecco’s Modified Eagle Medium (DMEM, Gibco, Waltham, MA, USA) supplemented with 2% fetal bovine serum (FBS, OPCEL, Guangzhou, China), 100 IU/mL penicillin, and 100 µg/mL streptomycin, and maintained at 37 °C in a humidified 5% CO_2_ incubator. When PK-15 and ST cells reached approximately 70–80% confluence in 6-well plates, the growth medium was aspirated, and the cell monolayer was gently washed twice with sterile PBS. Subsequently, 200 µL of the filtered fecal supernatant was inoculated onto the cell monolayer; uninoculated cells served as negative controls. Inoculated cells were incubated at 37 °C for 2 h to allow virus adsorption, with gentle rocking of the flasks/plates every 15 min to ensure even coverage. Following adsorption, the inoculum was removed, and cells were overlaid with maintenance DMEM containing 2% FBS and the aforementioned antibiotics. Inoculated PK-15 and ST cells were examined daily under an inverted light microscope for the development of CPE. If no CPE was observed, cell culture supernatant was collected for viral identification.

Detection and identification of PAdV-5 (Supplementary Table S1) were performed using polymerase chain reaction (PCR) targeting a specific region of the virus. An amount of 200 µL of cell culture supernatant showing CPE or from the final passage was used for nucleic acid extraction. Total nucleic acid was extracted using a commercial viral DNA/RNA extraction kit (Omega Bio-tek, Norcross, GA, USA). Specific primers targeting conserved regions of PAdV-5 (hexon gene) were designed and synthesized for PCR amplification. PCR products were analyzed by electrophoresis on a 2% agarose gel (Sangon Biotech) stained with nucleic acid dye (TransGen Biotech, Beijing, China), and bands of the expected size were visualized under UV light using a gel documentation system (Tanon, Shanghai, China) to confirm the presence of PAdV-5. Positive PCR products from isolates could be further sequenced for genotypic confirmation.

### 2.2. Genomic Sequencing and Analysis

Viral genomic DNA from PCR-confirmed PAdV-5 isolates was extracted from infected cell culture supernatants exhibiting significant CPE using a commercial viral DNA extraction kit. DNA concentration and purity were assessed fluorometrically. The complete genome of PCR-confirmed PAdV-5 isolates was amplified in 15 overlapping fragments using virus-specific primers. Each fragment (1.5–3.0 kb) shared ≥200 bp overlaps with adjacent segments to ensure full genome coverage and mitigate primer-derived sequencing errors. PCR products were purified (PCR Purification Kit, Omega Bio-tek, Norcross, GA, USA), ligated into the pMD19-T vector (Takara Bio, Kusatsu, Japan) and transformed into competent *Escherichia coli* (*E. coli*) DH5α cells (Tiangen Biotech, Beijing, China). Positive clones were screened by colony PCR and plasmid extraction (Plasmid Mini Kit, Omega Bio-tek). Purified recombinant plasmids were submitted to Sangon Biotech for bidirectional Sanger sequencing using universal M13 primers. To further confirm the viral genome sequence, whole-genome sequencing was performed by Sangon Biotech using next-generation sequencing (NGS) platforms. Raw sequencing reads were assembled using SPAdes, and the dominant contig was identified as PAdV-5 by BLASTn against the NCBI database (reference: AF289262). Genome termini were validated by Sanger sequencing of PCR-amplified ITR junctions. The complete genome was annotated using NCBI ORFfinder and Vgas with manual curation based on PAdV-5 reference genes.

Based on PAdV-5 gene functions (hexon, fiber), complete genomes of reference porcine mastadenoviruses were aligned with MUSCLE and A maximum-likelihood phylogenetic tree was constructed in **MEGA** using the TN93+G+I model [17].

### 2.3. Construction of Infectious Clone

The full-length infectious clone of porcine adenovirus type 5 (PAdV-5) was engineered using Red/ET recombineering in *Escherichia coli* GBred-gyrA462, which induces the RecET operon with L-arabinose (Sangon Biotech). Direct cloning of the viral genome was achieved via linear-to-linear homologous recombination (LLHR) [18]. First, intact PAdV-5 genomic DNA was isolated from CsCl-gradient-purified virions (CsCl, Sigma-Aldrich, St. Louis, MO, USA) using a proteinase K/SDS-phenol-chloroform protocol to prevent DNA shearing. Concurrently, the p15A-Amp-ccdB vector backbone was amplified via PCR with primers containing 50-nt homology arms (HAs) complementary to the PAdV-5 inverted terminal repeats (ITRs). The purified viral DNA (500 ng) and linearized backbone (500 ng) were co-electroporated into L-arabinose-induced *E. coli* GB05-dir. Recombinants were selected on ampicillin (Sangon Biotech) plates, confirmed by PCR and restriction analysis, yielding the full-length clone p15A-PAdV5-Full within 1 week.

To generate four E3-modified PAdV-5 variants, site-specific homology arms (HAs) were designed for each locus (Supplementary Table S1). The GFP-kan-ccdB cassette was amplified from plasmid pR6K-mCMV-eGFP-SV40-kanccdB using four primer pairs containing HAs, followed by co-electroporation with p15A-PAdV5-Full into L-arabinose-induced *E. coli* GBRed-GyrA462 (Redαβγ, gyrA462 mutant). Recombinants were selected on kanamycin (50 µg/mL) + ampicillin (50 µg/mL) plates, yielding intermediate clones p15A-PAdV5-E3[A-D]-mCMV-eGFP-SV40-kanccdB. For scarless removal of the kanamycin-ccdB element, each plasmid was linearized by I-SceI digestion (New England Biolabs, Ipswich, MA, USA), then co-electroporated with a corresponding 90-nt single-stranded oligonucleotide (Sangon Biotech) into *E. coli* GB05-dir. Through RecET-mediated linear-to-linear homologous recombination, the oligonucleotides bridged the I-SceI termini via 45 nt overlaps, precisely excluding kan-ccdB while preserving the GFP unit.

### 2.4. Virus Rescue

The reconstitution of recombinant PAdV-5 was initiated by digesting p15A-PAdV5-E3[A-D]-mCMV-eGFP-SV40 plasmids with FseI, which cleaves pre-engineered sites flanking the viral genome. Following incubation at 37 °C for 4 h (5 U/μg DNA), reactions were terminated by adding 0.1 vol 3 M sodium acetate (pH 5.2) and 2.5 vol ice-cold absolute ethanol. DNA was precipitated at −20 °C for 1 h, pelleted by centrifugation (12,000× *g*, 30 min, 4 °C), washed twice with 70% ethanol, and air-dried. Pellets were gently resuspended in nuclease-free water to 500 ng/μL, with linearization efficiency confirmed by 0.8% agarose gel electrophoresis. For transfection, ST cells (swine testicular cell line) were seeded in 6-well plates (5 × 10^5^ cells/well) and grown to 90% confluence. A total of 2 μg linearized DNA was mixed with 5 μL Lipofectamine 3000 (Invitrogen, Waltham, MA, USA) in Opti-MEM (Gibco), incubated for 15 min at RT, and added to cells. After 6 h, the medium was replaced with maintenance DMEM (2% FBS). Cells were monitored daily for GFP fluorescence and Cytopathic effect [19]. Following transfection of linearized recombinant plasmids into ST cells (F0), successful rescue was confirmed by GFP fluorescence at 72 h post-transfection. Rescued viruses were subjected to three serial passages (F1–F3) in ST cells under standardized conditions. At passage 5 (F5), recombinant viruses (ΔE3-12.5K-GFP, ΔE3-12.5K+ORF4-GFP, E3-Insert-GFP) and mock-infected controls were inoculated onto ST cell monolayers in 24-well plates at MOI = 1. Quantitative analysis was performed on a web-based image analysis platform (https://ij.imjoy.io/, accessed on 16 September 2025) through measurement of corrected total cell fluorescence (CTCF) calculated as CTCF = Integrated Density − (Area of selected region × Mean background intensity), where background values derived from mock-infected controls. Three biological replicates were analyzed, with statistical significance determined by one-way ANOVA followed by Tukey’s multiple comparisons test.

### 2.5. Viral Titration and Replication Kinetics Analysis

Viral titers were determined using the 50% tissue culture infectious dose (TCID_50_) endpoint dilution method as described by Reed and Muench [20]. Briefly, ST cells were seeded into 96-well plates at 2 × 10^4^ cells/well and incubated overnight to form monolayers. Ten-fold serial dilutions (10^−2^ to 10^−9^) of virus stocks were prepared in DMEM with 2% FBS, with eight replicate wells inoculated per dilution. After 5 days incubation at 37 °C/5% CO_2_, endpoints were assessed: wild-type virus by cytopathic effect observation, and GFP-expressing recombinants by fluorescence detection at 488 nm. Titers were calculated using the Reed-Muench formula [21].

Medium (DMEM) supplemented with 10% fetal bovine serum at 37 °C with 5% CO_2_. Cells were infected with recombinant PAdV-5 strains (N1, N3, P1, P3, Q1, Q3) at an MOI of 1. Mock controls received virus-free medium.

For one-step growth curve analysis [22], ST cell monolayers in 12-well plates were infected at a multiplicity of infection (MOI) of 0.1 in triplicate. Following 1 h adsorption at 37 °C with gentle rocking every 15 min, the inoculum was removed, cells were washed twice with phosphate-buffered saline (PBS), and overlaid with 1.5 mL maintenance medium (DMEM + 2% FBS). Samples were harvested at 12 h intervals from 0 to 72 h post-infection (hpi) and 24 h intervals from 72 to 168 encompassing 10 time points (0, 12, 24, 36, 48, 60, 72, 96, 120, 144, 168 hpi). At each collection point, entire wells were subjected to triple freeze–thaw cycling (−80 °C/37 °C), followed by centrifugation at 3000× *g* for 10 min to clarify supernatants. Viral genome quantification was performed by qPCR using previously validated methods. Group comparisons were performed using Kruskal–Wallis non-parametric ANOVA followed by Dunn’s multiple comparisons test (n = 3). A probability value of *p* < 0.05 was considered statistically significant.

### 2.6. Quantitative Analysis of GFP Fluorescence

GFP fluorescence images were captured 48 h post-infection using a microscope equipped with a 20× objective lens and standard GFP filter set (excitation 470/40 nm, emission 525/50 nm). All images were acquired with identical exposure times (500 ms) and gain settings (2×) to ensure comparability between samples. For each virus strain and mock control, six random fields of view were captured from three independent biological replicates.

Fluorescence quantification was performed using ImageJ software (NIH, version 1.53k). Raw images were converted to 8-bit grayscale prior to analysis. For each field of view, the entire infected area was selected using the freehand selection tool, ensuring inclusion of all GFP-positive cells while excluding obvious artifacts or non-infected areas. Three cell-free regions adjacent to the infected area were selected for background measurement.

The specific total fluorescence was calculated using the formula:

Spec_IntDen = IntDen − (Mean_background × Area) where IntDen represents the integrated density of the selected area, Mean_background is the average mean gray value from three background measurements, and Area is the pixel area of the selected region. Negative values resulting from background subtraction were set to zero to reflect biological reality.

All statistical analyses were performed using GraphPad Prism 9.0. Data are presented as mean ± standard deviation of at least six independent fields of view from three biological replicates. Group comparisons were performed using Kruskal–Wallis non-parametric ANOVA followed by Dunn’s multiple comparisons test. A probability value of *p* < 0.05 was considered statistically significant.

## 3. Results

### 3.1. Epidemiology and Cell Line-Dependent Virus Isolation

PCR screening of 167 clinical samples (feces, spleen, and anal swabs) across Hunan and Guangdong provinces (November 2021–March 2022) revealed a 38.3% (64/167) PAdV-5 positivity rate, with notable epidemiological variations. As summarized in Table 1, spleen samples exhibited the highest detection rate (64.7%, 11/17), particularly from Guangdong diarrheic cases, while anal swabs from Hunan showed 47.8% positivity (11/23).

**Figure 1:**
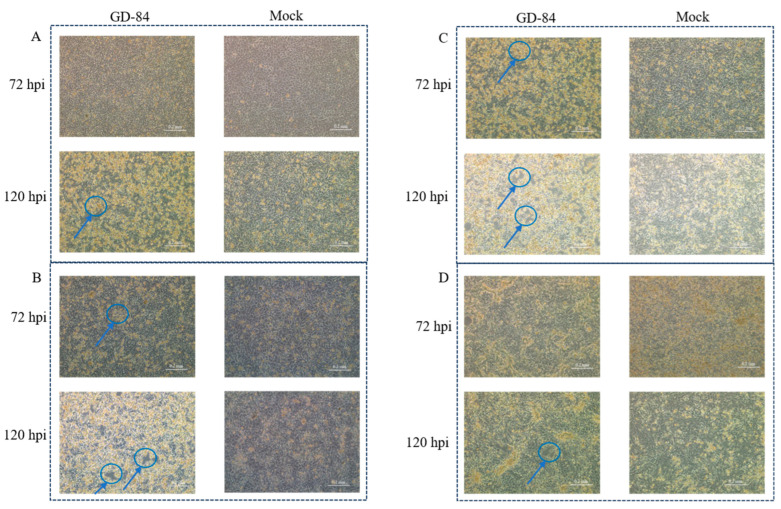
Temporal progression of CPE during PAdV-5 GD84 isolation and adaptation. (**A**) ST cells at F3 passage: Mock (right) and Infected (left) at 72 hpi (top) and 120 hpi (bottom). (**B**) ST cells at F5 passage. (**C**) ST cells at F15 passage (**D**) PK-15 cells at F15 passage (scale bar: 200 μm). All images: arrows indicate representative cells exhibiting characteristic adenoviral CPE, including cell rounding, aggregation, or increased granularity. 10% spleen homogenate inoculum from sample #84. Representative of n = 3 experiments.

Inoculation of PCR-positive samples onto PK-15 and ST cells showed no cytopathic effects during the first two blind passages (F1-F2), despite persistent viral DNA detection. A critical breakthrough occurred at F3 passage in ST cells inoculated with splenic sample GD84 (Guangdong 2021), where distinct CPE—characterized by cell rounding, aggregation, and granularity—emerged at 120 h post-inoculation (Figure 1A). Following isolation, serial propagation in ST cells demonstrated progressive adaptation: F5 passage exhibited CPE at 72 hpi (Figure 1B), while F15 passage showed complete monolayer disruption at 72 hpi (Figure 1C). Notably, parallel passage in PK-15 cells to F15 revealed significantly attenuated CPE development at 72 hpi compared to ST cells (Figure 1D), confirming cell line specificity. Quantitative titration from F5 to F15 revealed viral titers plateaued at ~10^7.5^ TCID_50_/mL, indicating stable replication efficiency without significant increase beyond passage 5. Genetic stability was confirmed through consistent hexon gene amplification across passages (F5/F10/F15) and 100% nucleotide identity in sequencing.

### 3.2. Genomic Characterization and Evolutionary Analysis of PAdV-5 GD84

The complete genome of isolate Mastadenovirus porcusquintum strain GD84/China/2024 (PAdV-5-GD84, F5 and F10 passage) was sequenced using Illumina NovaSeq 6000. De novo assembly yielded a linear dsDNA molecule of 32,620 bp, with 147 bp inverted terminal repeats (ITRs) and a GC content of 51.0%, notably lower than the *Mastadenovirus* genus average (53–55%) yet exhibiting striking evolutionary relationships:

Whole-genome homology:

99.0% nt identity to contemporary strain PAdV-5/JP/2020 (LC-702314)

97.0% nt identity to prototype PAdV-5-HNF-70 (AF289262)

**Critical gene divergence:**

**Gene**

**vs. LC-702314**

**vs. AF289262**

**Functional Implication**

***Hexon***

**99.38%**

**89.00%**

**Altered neutralizing epitopes**

***Fiber***

**98.32%**

**90.30%**

**Modified receptor tropism**



**Figure 2:**
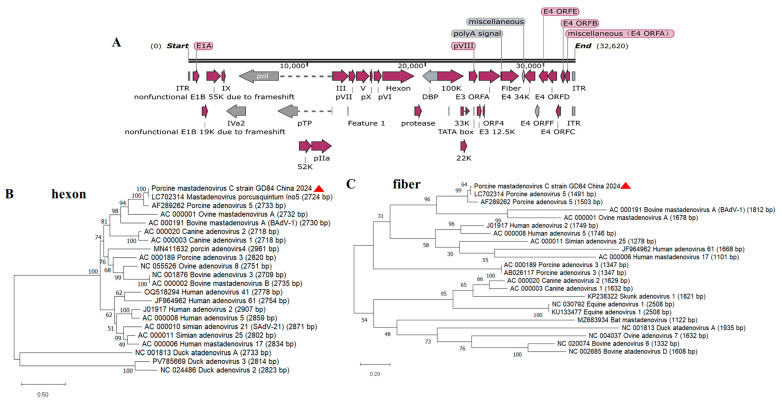
Genome organization of Mastadenovirus porcusquintum strain GD84/China/2024 (**A**) and phylogenetic analysis based on the full genome nucleotide sequences of the hexon (**B**) and iber (**C**).

This near-identical genome length but reduced GC content suggests large-scale nucleotide substitutions rather than deletions. Strikingly, extreme divergence in structural genes. Open reading frames were predicted by SnapGene. Despite near-complete synteny with the prototype AF289262 across its 32,620 bp architecture, targeted annotation revealed critical modifications in functional domains—particularly within early transcriptional units governing host adaptation. The canonical adenoviral genome organization was conserved (Figure 2A), including Early Regions (E1–E4), Late Regions (L1–L5), and Non-coding Elements (ITRs), yet key mutations implicated altered virulence and immune evasion strategies.

Maximum-likelihood analysis demonstrated distinct evolutionary patterns between structural genes: PAdV-5-GD84’s hexon gene (2724 bp) formed a monophyletic clade with contemporary porcine adenoviruses LC702314 and prototype AF289262 (100% bootstrap support), yet exhibited significant affinity with ruminant adenoviruses through an 81%-supported node linking to ovine (AC_000001) and bovine (AC_000191) strains—suggesting historical cross-species transmission (Figure 2B). In stark contrast, the fiber gene (Figure 2C) showed exclusive porcine clustering with 100% bootstrap support for GD84-LC702314-AF289262, completely segregating from non-porcine lineages including bovine adenoviruses (<65% bootstrap to BAdV-1) and primates (76%-supported human/simian clade). This topological discordance (Kishino–Hasegawa *p* = 0.002) was quantified by elevated evolutionary distance in hexon versus fiber (15.2% vs. 8.7% nt divergence from BAdV-1), with hexon’s ruminant association potentially originating from ancestral recombination while fiber maintained strict host fidelity through strong purifying selection (ω = 0.03 vs. hexon’s ω = 0.92).

### 3.3. Generation and Validation of Recombinant PAdV-5 Genomes with Targeted E3-mCMV-eGFP-SV40 Modifications

**Figure 3:**
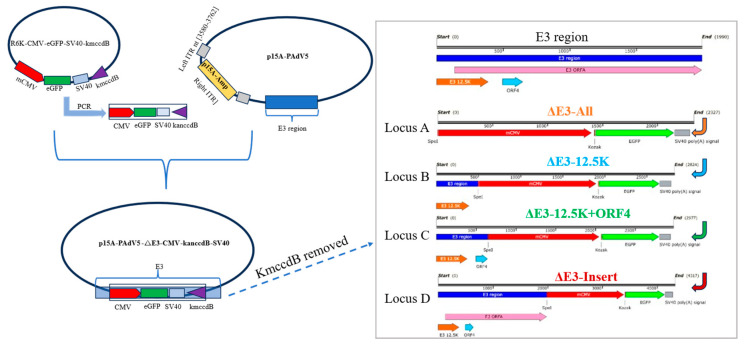
Schematic diagram of the construction strategy for E3-deleted infectious clones.

Four recombinant PAdV-5 genomes harboring distinct modifications within the E3 region and expressing eGFP were successfully constructed (Figure 3).

A. ΔE3-All-mCMV-eGFP-SV40: Complete replacement of the E3 region (nt [0–1990]) with the mCMV-eGFP-SV40 polyA cassette.
B. ΔE3-12.5K-mCMV-eGFP-SV40: Deletion of E3 sequences flanking the 12.5K ORF (nt [498–1990]), with retention of the intact 12.5K ORF and insertion of the GFP cassette adjacent to it.
C. ΔE3-12.5K+ORF4-mCMV-eGFP-SV40: Deletion of intervening E3 sequences between the preserved 12.5K ORF and ORF4 (nt [651–1990]), with insertion of the GFP cassette.
D. E3-Insert-mCMV-eGFP-SV40: Insertion of the GFP cassette into the native E3 locus between ORFA and fiber (nt [1990]) without nucleotide deletion.

Molecular validation confirmed the fidelity of all four recombinant infectious clones (pPAdV5-ΔE3-All-mCMV-eGFP-SV40, pPAdV5-ΔE3-12.5K-mCMV-eGFP-SV40, pPAdV5-ΔE3-12.5K+ORF4-mCMV-eGFP-SV40, pPAdV5-E3-Insert-mCMV-eGFP-SV40). Diagnostic PCR and Sanger sequencing across the modified loci verified precise genomic structures, including intended deletions, intact preservation of specified ORFs (12.5K, ORF4), and correct insertion of the intact GFP expression cassette.

### 3.4. Rescue Efficiency and Transgene Stability of Recombinant Viruses

**Figure 4:**
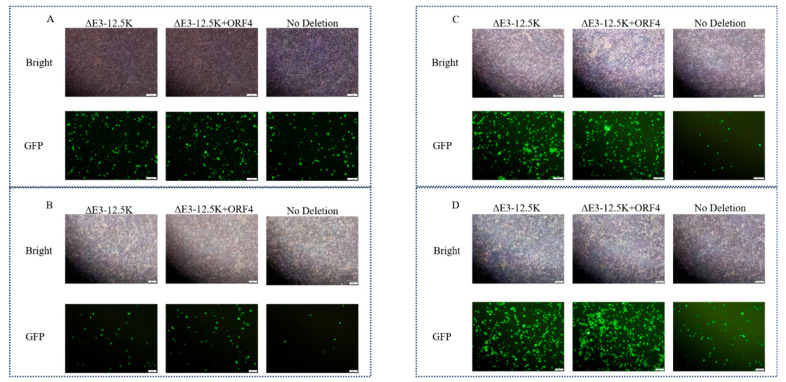
Serial passage monitoring of recombinant PAdV-5 viruses from F0 to F3. (**A**) F0 (72 h post-transfection) Representative merged images of ST cells transfected with Left: ΔE3-12.5K-GFP, Center: ΔE3-12.5K+ORF4-GFP, Right: E3-Insert-GFP (**B**) F1 (72 hpi) First virus passage. (**C**) F2 (72 hpi) Second passage. (**D**) F3 (72 hpi) Third passage. Scale bars: 100 μm (all panels).

Consistent with the functional requirement of the E3-encoded 12.5K ORF, no infectious virus was recovered from ΔE3-All-mCMV-eGFP-SV40 transfections across three independent trials. In stark contrast, robust GFP expression confirmed successful rescue of E3-Insert-mCMV-eGFP-SV40 (full E3 + insertion), ΔE3-12.5K-mCMV-eGFP-SV40 (12.5K preserved), ΔE3-12.5K+ORF4-mCMV-eGFP-SV40 (12.5K+ORF4 preserved), and wild-type PAdV-5 controls, unequivocally establishing 12.5K as indispensable for PAdV-5 replication. Subsequent characterization of ΔE3-12.5K-eGFP, ΔE3-12.5K+ORF4-eGFP, and E3-Insert-eGFP constructs revealed robust eGFP fluorescence at 72h post-transfection (Figure 4A), with sustained expression persisting through F1-F3 serial passages (Figure 4B,D).

**Figure 5:**
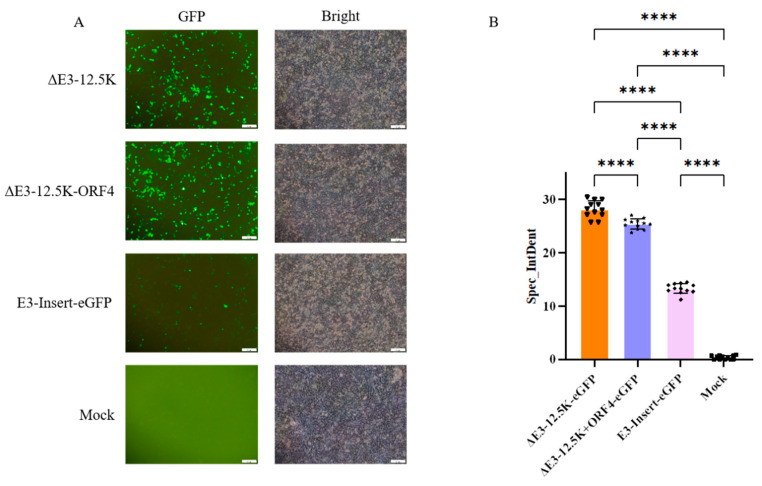
Quantitative comparison of fluorescence intensity in F5-passaged recombinant viruses. (**A**) Representative fluorescence images of ST cells infected with recombinant PAdV-5 variants at MOI = 1 (72 hpi). Scale bars: 100 μm (**B**) Corrected total cell fluorescence (CTCF) analysis. Data shown as mean. **** *p* < 0.001.

All recombinants exhibited progressive cytopathic effects (Supplementary Figure S1), and maintained stable reporter expression over multiple replication cycles, confirming preservation of replicative fitness. Notably, the full-length E3-Insert-eGFP consistently demonstrated attenuated fluorescence intensity compared to ΔE3 variants (Figure 5), revealing an inverse relationship between genomic payload and expression efficiency that informs future vector design strategies.

### 3.5. One-Step Growth Curve Analysis

**Figure 6:**
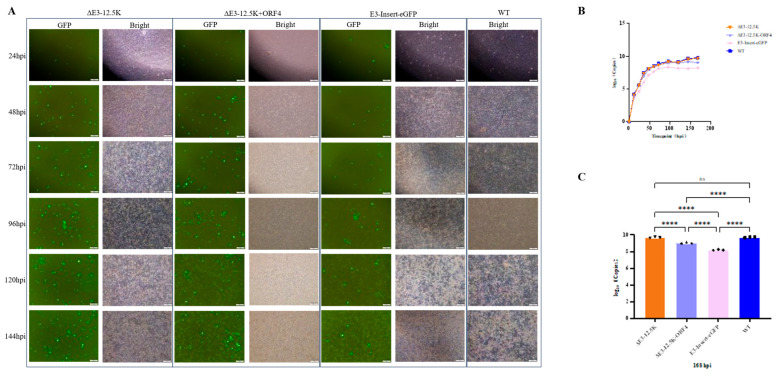
Comparative growth kinetics of PAdV-5 variants. (**A**) Time-course imaging, (scale bar: 100 μm). (**B**) Growth curves, line graph of viral titers (log_10_ copies/mL) from 0 to 168 hpi (n = 3). (**C**) Terminal titer comparison, bar chart at 168 hpi with asterisks indicating significance (**** *p* < 0.0001).

Following inoculation at MOI = 0.1, ST cells infected with recombinant viruses exhibited progressive cytopathology characterized by peak GFP fluorescence intensity at 96 hpi and widespread CPE exceeding 80% monolayer destruction by 120 hpi, coinciding with advanced syncytia formation and cell detachment across all strains (Figure 6A). Quantitative growth kinetics revealed that while wild-type virus (WT) and ΔE3-12.5K achieved high terminal titers of 10^9.72^ and 10^9.63^ copies/mL, respectively, at 168 hpi with near-identical biphasic growth profiles (Figure 6B). Terminal comparison at 168 hpi confirmed statistically equivalent yields for WT and ΔE3-12.5K (*p* = 0.8298), but profound attenuation for E3-Insert-eGFP (<0.01% of WT, *p* < 0.0001) and a significant reduction for ΔE3-12.5K+ORF4 (19% of WT, *p* < 0.0001). This demonstrates that exogenous gene insertion imposed the greatest replicative burden, despite this construct inducing maximal fluorescence at 96 hpi (Figure 6C).

## 4. Discussion

Domestic pigs and wild boars are currently widely recognized as the natural hosts for PAdV-5 [23]. Related studies also indicate that PAdV-5 specifically infects only certain porcine-related cells. Therefore, our virus isolation efforts were primarily conducted on ST and PK cell lines. Based on factors such as CPE, time to CPE onset, and cell culture maintenance time, the ST cell line was selected for viral isolation and rescue. In this study, a PAdV-5 was isolated from diarrheic piglets. Full-length genome was 32,620 bp. The viral genome was determined via NGS. Nucleotide sequence alignment and phylogenetic analysis demonstrated that it belongs to a strain of PAdV-5. Based on previously published genomic data, only two complete genomes (HNF-70 and Ino5) of PAdV-5, along with several incomplete viral genomes, are currently publicly available. While currently available data provide initial insights, comprehensive PAdV characterization will require substantially broader sequence coverage and integrated bio-informatics analyses. Consequently, PAdV-5 was isolated and a reverse genetics platform established to facilitate viral vector vaccine development.

Since the first successful isolation of porcine adenovirus (PAdV) in 1964, multiple serotypes have been identified and extensively characterized, with type 3 (PAdV-3) and type 5 (PAdV-5) emerging as research priorities due to their notable pathogenicity. Although the PAdV-5 strain in this study was isolated from diarrheic piglets, and peritubular nephritis has been previously associated with porcine adenovirus infection, the source animals exhibited no clinical signs of renal, respiratory, or enteric disease. This aligns with current epidemiological consensus that PAdVs frequently establish subclinical infections in swine populations, with disease manifestation likely requiring cofactors such as immunosuppression, concurrent pathogens, or stress triggers [5,24]. Critically, no direct causal relationship has been substantiated between PAdV-5 and specific clinical syndromes including diarrhea, respiratory disease, nephritis, or enteritis. The virus’ detection in symptomatic individuals may thus represent opportunistic replication rather than primary pathogenesis—a phenomenon documented for multiple adenoviruses across species. While capable of causing fatal respiratory disease in immunocompromised hosts [25], most HAdV infections are subclinical—enabling their widespread use as vaccine/gene therapy vectors [26,27]. Thus, PAdV-5’s stable swine-specific tropism and absence of zoonotic reports suggest enhanced safety for veterinary vector development.

The complete genome of PAdV-GD84 (this study) measures 32,620 bp, closely matching the size of strain HNF-70 (32,621 bp). However, whole-genome sequence alignment revealed higher nucleotide identity between PAdV-GD84 and Ino5 (99.0%) compared to HNF-70 (97.0%). The overall GC content of PAdV-GD84 was 51.0%, slightly lower than other PAdV-5 strains (53.0%). Phylogenetic analyses of both hexon and fiber genes further demonstrated that while PAdV-GD84, Ino5, and HNF-70 cluster together, PAdV-GD84 exhibits closer genetic relationship with Ino5. This apparent genomic paradox—conserved length with divergent identity—likely stems from recombination-mediated modular evolution, where homologous exchange between circulating strains replaces genomic segments while preserving architecture [23]. The reduced GC content (51% vs. genus average 53–55%) may reflect adaptive nucleotide bias in response to host immune pressures, particularly given elevated nonsynonymous substitutions in hexon (dN/dS = 1.2 vs. fiber’s 0.3). Notably, GD84’s closer relationship to contemporary Ino5 (JP/2020) than to historical HNF-70 (1987) suggests spatiotemporal divergence within porcine mastadenovirus C, potentially driven by regional transmission bottlenecks [28].

The reverse genetics platform developed here addresses two major constraints in PAdV-based vector design: construction efficiency and pre-existing immunity. Firstly, our Red/ET system enables the rapid generation of infectious clones within three weeks, significantly outperforming traditional time-consuming methods reliant on scarce restriction sites or BAC systems [18]. More importantly, this efficiency allows for the strategic exploration of solutions to the second challenge: the high seroprevalence of PAdV-5 antibodies in swine herds (38.3%, this study), which could neutralize vaccine vectors based on homologous strains. Our platform is ideally suited to rapidly engineer vectors based on prime-boost regimens or to create chimeric vectors with swapped capsid proteins to evade neutralization, strategies proven effective in human adenovirology [26]. Furthermore, the contemporary GD84 strain characterized here may itself offer an advantage over historical laboratory strains (e.g., HNF-70), as vectors derived from currently circulating viruses might experience less pre-existing immunity in the field.

In constructing replication-competent adenoviral vectors, the primary strategy involves partial deletion of the non-essential E3 region while maintaining the integrity of the E1 region [29]. This presents a critical balance in developing PAdV-5-based replicating vector vaccines: accommodating large foreign gene inserts while preserving viral replication competence and passage stability. Previous studies have identified ORF2 (the 12.5K ORF in this study) as essential for successful PAdV-5 rescue [16]. Our findings corroborate this observation: while ΔE3-All-eGFP failed to be rescued (Supplementary Figure S2), both ΔE3-12.5K-eGFP and ΔE3-12.5K+ORF4-eGFP constructs, along with E3-Insert-eGFP, were successfully rescued and demonstrated stable passage. Notably, E3-Insert-eGFP exhibited significantly lower fluorescence intensity and total expression during rescue and passage compared to ΔE3-12.5K-eGFP and ΔE3-12.5K+ORF4-eGFP, suggesting that genomic size critically impacts viral replication—an oversized genome imposes substantial replicative burden. For E3 region replacement strategies, we selected ΔE3-12.5K-eGFP as the optimal construct because it deletes 62.46% of the E3 region (reducing genome size), preserving the essential 12.5K ORF and simultaneously satisfies both replicative requirements and capacity for large foreign gene insertion.

The combination of our reverse genetics platform and the GD84 strain offers a compelling vector system for veterinary vaccine development. Firstly, the efficiency of the platform allows for rapid iteration and screening of vaccine candidates. Secondly, the functional mapping of the E3 region provides a validated blueprint for inserting exogenous antigens without compromising viral fitness. Finally, and crucially, GD84 is a contemporary strain isolated from a major swine-producing region. Vectors based on such circulating strains may offer practical immunogenic advantages over those based on historical, laboratory-adapted strains, potentially mitigating the impact of pre-existing immunity in target animal populations—a known challenge for adenoviral vectors.

In summary, this study not only isolates and characterizes a contemporary PAdV-5 strain but also delivers a versatile and efficient reverse genetics platform. We further developed a versatile reverse genetics platform for PAdV-5, which enabled the generation of replication-competent recombinant viruses through targeted replacement of the E3 region with an eGFP reporter cassette. The resulting infectious clones not only provide crucial tools for investigating PAdV-5 pathogenesis but also establish a foundational platform for multiple applications: they will accelerate vaccine development by enabling rapid antigen screening, facilitate antiviral drug discovery through direct viral replication monitoring, support serological surveillance through standardized antibody detection, and offer a strategic blueprint for adapting this system to other respiratory adenoviruses. Importantly, the successful maintenance of viral replication capacity while accommodating foreign gene insertion demonstrates the potential for developing PAdV-5 as a vaccine vector for larger antigens.

## Figures and Tables

**Table 1:** PAdV-5 detection in Chinese swine (2021–2022).

Date	Sample Type	Source Location	Total Samples	Positive Samples	Detection Rate (%)	Clinical Notes
20 May 2021	Feces	Guangdong, China	30	1	3.3	SPF pigs
25 November 2021	Feces	Hunan, China	32	15	46.9	Normal
9 December 2021	Feces	Guangdong, China	20	4	20.0	Normal
20 December 2021	Spleen	Guangdong, China	17	11	64.7	Diarrhea observed
11 January 2022	Feces	Guangdong, China	45	22	48.9	Normal
1 March 2022	Anal swab	Hunan, China	23	11	47.8	Normal

Normal: no observable clinical signs at sampling.

## Data Availability

The raw sequencing data generated in this study are openly available in the NCBI Sequence Read Archive (SRA) repository under accession number PRJNA1308366.

## Supplementary Materials

The following supporting information can be downloaded at: https://www.mdpi.com/article/10.3390/v17091270/s1, Figure S1: Phenotypic analysis of recombinant PAdV-5 mutants over time; Figure S2: Serial passaging of rescued ΔE3-All-eGFP virus; Table S1: Primers used for recombinant adenovirus vector construction.
