# Supplementary material for: Isolation of Porcine Adenovirus Serotype 5 and Construction of Recombinant Virus as a Vector Platform for Vaccine Development
Source: Viruses. 2025 Sep 19;17(9):1270. doi: 10.3390/v17091270 (PMC12474365; doi:10.3390/v17091270)
Supplement: Supplementary file 1 [file viruses-17-01270-s001.zip › viruses-3858999-Supplementary Materials.pdf]

Supplement Data

Table S1. Primers used for recombinant adenovirus vector construction

Primer Name	Sequence (standard)	Purpose (Vector Construction)
mCMV-eGFP-F	CTCGCTGCAGGCGGCCGCTCTAGAACTCGTCGATGCCACCATGGTGAGCAAGGGCGAGGA	Construction of R6K-CMV-eGFP-SV40-kmccdB
SV40-eGFP-R	TTTGTAACCATTATAAGCTGCAATAAACAAGTTAATTCGTTACTTGTACAGCTCGTCCATGCCG	
R6K-SV40-F	CGAATTAACTTGTTTATTGCAGCTTATAATGG	
R6K-mCMV-R	GGTGGCATCGACGAGTTCTA	
PAV5-deleteE3(long)-GFP-F	CTACGACATAGTCTCTGACTCTGTCGACGGTTATGACTGATCTAATTAAAAATATCGTTTATTAGACTTGACTAG	p15A-PAdV5-ΔE3-All-mCMV-eGFP-SV40: Complete replacement of the E3 region (nt [28045-30027]) with the mCMV-eGFP-SV40 polyA cassette
PAV5-deleteE3（Long）-GFP-R	AAAAAGTCAGATGTATTTTTAGAATGCTTTATTAGTGCACCAATTACCCTGTTATCCCTAGCTTG	
p15A-PAV5-mCMV-F	GCCCGTCATGTGTACCACAGACAACCATCCCAGAGAAACGGCCACTACTAGTCGCGCCAACTCCG	p15A-PAdV5-ΔE3-12.5K-mCMV-eGFP-SV40: Deletion of E3 sequences flanking the 12.5K ORF (nt [28635-29872]), with retention of the intact 12.5K ORF and insertion of the GFP cassette adjacent to it
p15A-PAV5-km-R	ATTACCATGATATACATATAATCATTTTTTTGATCAAATGAATTAATTACCCTGTTATCCCTAGCTTGCA	
p15A-PAV5-E3-ORF4-mCMV-F	TTCGAGACCACTGCAACTTTCCACCGACCGTTAGATACATGCTGAACTAGTCGCGCCAACTCCGC	p15A-PAdV5-ΔE3-12.5K+ORF4-mCMV-eGFP-SV40: Deletion of intervening E3 sequences between the preserved 12.5K ORF and ORF4 (nt [28688-29872]), with insertion of the GFP cassette
p15A-PAV5-km-R	ATTACCATGATATACATATAATCATTTTTTTGATCAAATGAATTAATTACCCTGTTATCCCTAGCTTGCA	
PAV5-completeE3-PolyA-mCMV-F	GGCAAACATTTTGATGATGATGATGTCTAATGGTGCACTAATAAAACTAGTCGCGCCAACTCCGC	p15A-PAdV5-D. E3-Insert-mCMV-eGFP-SV40: Insertion of the GFP cassette into the native E3 locus between ORFA and fiber (nt [30041]) without nucleotide deletion.
PAV5-completeE3-Fiber-Km-R	CGCGTTTCATGCTAAAACAAAAAGTCAGATGTATTTTTAGAATGCATTACCCTGTTATCCCTAGCTTGCAG	
Detection of PAV5-F	TACTGCAAGTTCCACATCCAGGT	Detection of PAdV-5
Detection of PAV5-R	GGAATGGAGATGGGCAGGTT	
Hexon-cut-F	GATGTCATGGACAACGTCAAC	
Hexon-cut-R	CACGGAGGAGTCGAACTGGATG	

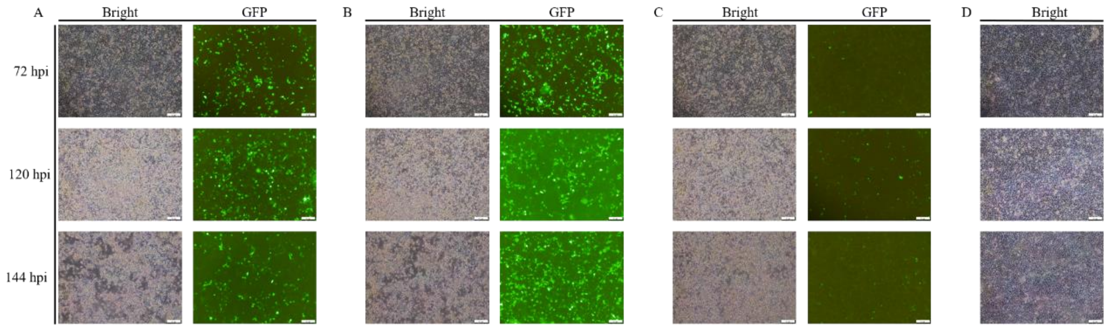

Figure S1. Phenotypic analysis of recombinant PAdV-5 mutants over time. (A) ΔE3-12.5K mutant at 72, 120, and 144 hpi (hours post-infection). (B) ΔE3-12.5K+ORF4 mutant. (C) E3-Insert-eGFP recombinant virus. (D) Mock-infected control. Scale bars: 100 μm (all panels).

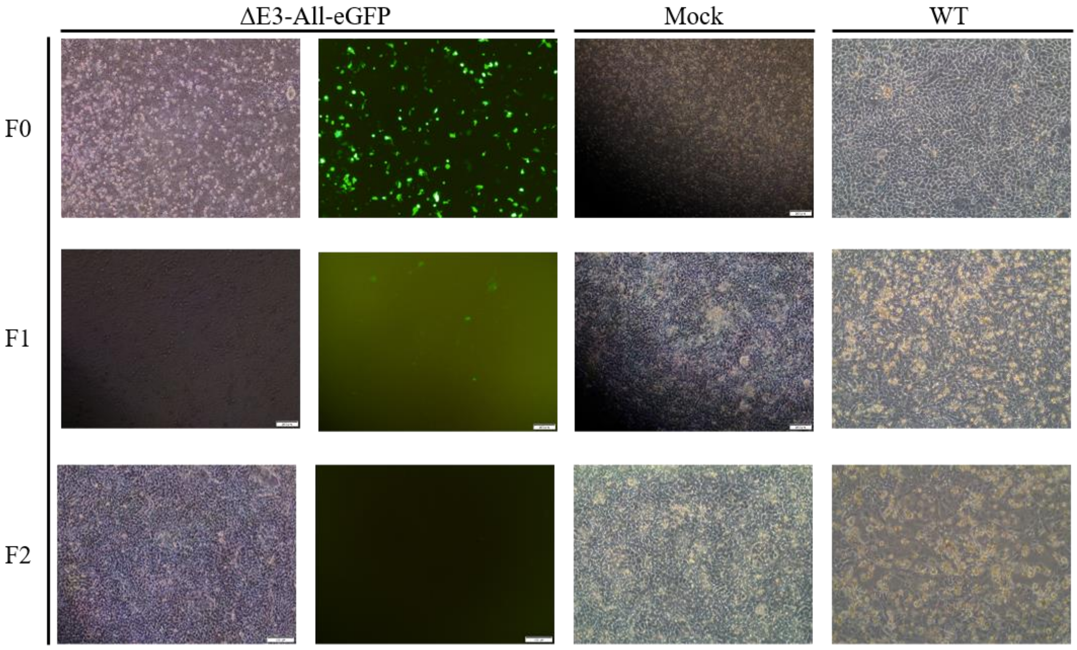

Figure S2. Serial passaging of rescued ΔE3-All-eGFP virus. ΔE3-All-eGFP: Recombinant virus with the entire E3 region deleted and replaced by the eGFP reporter gene. Mock: Mock infection (negative control), no virus inoculated. WT (Wild-Type): Wild-type virus control. The viral genome is identical to the parental wild-type strain with no gene replacements or insertions. Scale bars: 100 μm (all panels).
